# Molecular signatures of angiogenesis inhibitors: a single-embryo untargeted metabolomics approach in zebrafish

**DOI:** 10.1007/s00204-023-03655-5

**Published:** 2024-01-29

**Authors:** Pia Wilhelmi, Volker Haake, Franziska M. Zickgraf, Varun Giri, Philipp Ternes, Peter Driemert, Julia Nöth, Stefan Scholz, Marta Barenys, Burkhard Flick, Barbara Birk, Hennicke Kamp, Robert Landsiedel, Dorothee Funk-Weyer

**Affiliations:** 1 BASF SE, Experimental Toxicology and Ecology, Carl-Bosch-Strasse 38, 67056 Ludwigshafen Am Rhein, Germany; 2 University of Barcelona, Research Group in Toxicology-GRET, 08028 Barcelona, Spain; 3 BASF Metabolome Solutions, 10589 Berlin, Germany; 4 Department of Bioanalytical Ecotoxicology, Helmholtz Centre for Environmental Research-UFZ, 04318 Leipzig, Germany; 5 German Centre for the Protection of Laboratory Animals (Bf3R), German Federal Institute for Risk Assessment (BfR), 10589 Berlin, Germany; 6 Institute of Pharmacy, Pharmacology and Toxicology, Free University of Berlin, 14195 Berlin, Germany; 7 Present Address: Preclinical Compound Profiling, Toxicology, NUVISAN ICB GmbH, 13353 Berlin, Germany

**Keywords:** Untargeted metabolomics, Zebrafish embryo, Developmental toxicity, Angiogenesis

## Abstract

Angiogenesis is a key process in embryonic development, a disruption of this process can lead to severe developmental defects, such as limb malformations. The identification of molecular perturbations representative of antiangiogenesis in zebrafish embryo (ZFE) may guide the assessment of developmental toxicity from an endpoint- to a mechanism-based approach, thereby improving the extrapolation of findings to humans. Thus, the aim of the study was to discover molecular changes characteristic of antiangiogenesis and developmental toxicity. We exposed ZFEs to two antiangiogenic drugs (SU4312, sorafenib) and two developmental toxicants (methotrexate, rotenone) with putative antiangiogenic action. Molecular changes were measured by performing untargeted metabolomics in single embryos. The metabolome response was accompanied by the occurrence of morphological alterations. Two distinct metabolic effect patterns were observed. The first pattern comprised common effects of two specific angiogenesis inhibitors and the known teratogen methotrexate, strongly suggesting a shared mode of action of antiangiogenesis and developmental toxicity. The second pattern involved joint effects of methotrexate and rotenone, likely related to disturbances in energy metabolism. The metabolites of the first pattern, such as phosphatidylserines, pterines, retinol, or coenzyme Q precursors, represented potential links to antiangiogenesis and related developmental toxicity. The metabolic effect pattern can contribute to biomarker identification for a mechanism-based toxicological testing.

**Supplementary Information:**

The online version contains supplementary material available at 10.1007/s00204-023-03655-5.

## Introduction

Embryonic development is based on finely tuned and temporally coordinated processes and is therefore highly susceptible to substance-induced disturbances (Ross et al. 2015) that can manifest as congenital malformations. One of these sensitive processes is angiogenesis. It refers to the formation of new blood vessels from preexisting vessels, which is essential for proper supply of oxygen and nutrients to the developing tissues and organs (Rouwkema and Khademhosseini 2016). There is growing evidence that impaired vascular development can lead to congenital malformations, such as short limbs or heart defects (Beedie et al. 2016; Therapontos et al. 2009). Consequently, understanding of antiangiogenic mechanisms and identification of corresponding molecular markers is of eminent importance for developmental toxicology.

To unravel molecular changes of antiangiogenesis in developmental toxicity, we tested four reference compounds with known (SU4312, sorafenib) or putative antiangiogenic effect (methotrexate, rotenone). SU4312 is a selective inhibitor of the vascular endothelial growth factor receptor-2 (VEGFR2), a main regulator of angiogenesis (Sun et al. 1998). The pathway of VEGFR2 interaction and a causal link to the emergence of developmental defects were described in the adverse outcome pathway 43 (Knudsen et al. 2023). Sorafenib is a multikinase inhibitor targeting VEGFR2 among others (Wilhelm et al. 2006). Similar to SU4312, sorafenib is a pharmaceutical developed for cancer therapy via inhibition of angiogenesis (Wilhelm et al. 2004). In contrast to SU4312, sorafenib received marketing approval, and thus has been tested for developmental toxicity in the compulsory test species. An increased incidence in fetal malformations and retardations were observed in rats and rabbits (EMA 2007). Methotrexate is a folate antagonist and commonly known teratogen (Hyoun et al. 2012). The *fetal methotrexate syndrome* comprises a variety of congenital malformations observed in humans, ranging from microcephaly to cardiovascular anomalies and limb defects (Verberne et al. 2019). Several modes of action (MoAs) have been proposed to precede these malformations, including antiangiogenesis. It has been hypothesized that methotrexate exhibits antiangiogenic properties due to its efficacy in treating inflammatory diseases, which was supported by a rabbit cornea model showing a decrease in vascularization upon topical application (Joussen et al. 1999). Also, in zebrafish and human endothelial cells, an antiangiogenic phenotype has been observed upon methotrexate exposure (Schoors et al. 2015; Sun et al. 2009). In contrast, no significant reduction of microvessel outgrowth was observed in a human placenta assay (Fiehn et al. 2005), rendering the antiangiogenic potential of methotrexate as inconclusive. Similar to methotrexate, rotenone was connected to a multitude of MoAs. Given that rotenone is primarily a mitochondrial respiratory chain complex I inhibitor (Palmer et al. 1968), the mechanism of toxicity is strongly driven by impairments of the energy metabolism and oxidative stress. In vivo studies have found reduced fetal weight and skeletal abnormalities in rats (EPA 1987), while in vitro tests have classified rotenone as a putative vascular disruptor (McCollum et al. 2017). Thus, SU4312 and sorafenib have clearly been shown to interfere with angiogenesis, whereas the role of angiogenesis for developmental toxicity of methotrexate and rotenone is elusive.

Currently, developmental toxicity is mostly assessed using rats and rabbits. Yet, the zebrafish embryo (ZFE) has emerged as a promising alternative model and has shown to induce an antiangiogenic phenotype following exposure to SU4312 (Nöth et al. 2024), sorafenib (Beedie et al. 2016), methotrexate (Sun et al. 2009), and rotenone (McCollum et al. 2017). A major challenge with alternative models remains the extrapolation of findings to humans, as both exposure scenario and morphology differ between zebrafish and humans. Hence, recent alternative approaches incorporate the molecular mechanism of toxicity that precedes the apical morphological manifestation. These mechanisms are often congruent across species, as they rely on conserved signaling pathways such as those of early vertebrate embryogenesis (Artavanis-Tsakonas et al. 1999; NRC 2000; Zinski et al. 2018).

Omics techniques, such as transcriptomics or metabolomics, have proven to provide detailed insights into molecular changes representative of toxicity mechanisms (Dimopoulou et al. 2017; Ramirez-Hincapie et al. 2023). The plasma metabolome of rats exposed to over 600 reference compounds (Sperber et al. 2019) was measured to establish a database that can identify various toxicological endpoints such as maternal, liver, or kidney toxicity (Keller et al. 2019; Mattes et al. 2014, 2013) and predict the toxicity of new compounds based on their metabolic signature (van Ravenzwaay et al. 2016). The approach was further extended to in vitro systems, demonstrating that different modes of liver toxicity can be distinguished based on their metabolome response in HepG2 cells (Ramirez-Hincapie et al. 2023). Furthermore, we previously demonstrated through targeted metabolomics in ZFE that metabolite changes provide a mechanistic link to thyroid-related developmental toxicity (Wilhelmi et al. 2023). However, two major limiting factors emerged from this study. First, while the rat plasma was measured from the individual rat, ZFEs are mostly pooled for metabolome analyses to obtain a sufficient amount of biomass (Xu et al. 2023). Considering the heterogeneity of a treatment group, pooling might dilute relevant effects that occur only within a subset. Second, despite the targeted approach covered a wide range of biochemical pathways, important metabolites may still have been missed. Measurement of individual ZFEs could reduce the uncertainties associated with pooling, while enabling morphological assessment and metabolomics in the same individual. Untargeted metabolomics requires less biomass and aims to measure all metabolites that are technically feasible. We therefore used a single-embryo untargeted metabolomics approach to decipher molecular changes representative of antiangiogenesis and developmental toxicity based on the analysis of four reference compounds, SU4312, sorafenib, methotrexate, and rotenone.

## Material and methods

### Sample generation

Adult zebrafish (*Danio rerio*) from the in-house wild-type strain Obi/Wik (Helmholtz Centre for Environmental Research-UFZ Leipzig, Germany) were cultured according to German and European animal protection standards approved by the government of Saxony (Landesdirektion Leipzig, Germany, reference 75–9185.64). They were maintained on a day/night cycle of 14/10 h at 26 ℃. Spawning was initiated through light. Fertilized eggs in approximately eight-cell stage were collected and used for exposure to substances.

All reagents were purchased from Merck unless otherwise stated. The test substances had a minimum purity of 99%. Stock solutions of substances were prepared in DMSO and diluted using ISO water (according to OECD TG 236 (OECD 2013); 80 mM CaCl_2_·2H_2_O, 20 mM MgSO_4_·7H_2_O, 31 mM NaHCO_3_, 3.1 mM KCl, pH 7.4–7.5) to make up the required exposure concentration in 0.01% DMSO. The test substances SU4312 (CAS 5812-07-7), sorafenib (CAS 284461-73-0), rotenone (CAS 83-79-4), and methotrexate (CAS 59-05-2) were applied in three concentrations each (Table 1). The test concentrations were established based on lethal concentrations (LC_x_) derived from the preliminary concentration range-finding experiments. Lethality was assessed as described in OECD TG 236 (OECD 2013). The highest test concentration was chosen to be close to LC_20_ and the lowest to be around LC_1_, with an intermediate concentration chosen at appropriate spacing. Per test concentration, 16 embryos were treated individually in 96-well plates. Each treatment was performed on a separate plate. In addition, 48 embryos per plate were exposed to 0.01% DMSO in ISO water to obtain solvent control samples (plate layouts in supp. file4 Fig. S2). The exposure was conducted at 28 ± 1 ℃, from 3 to 4 h post-fertilization (hpf) until 96 hpf. Gross morphological alterations were assessed at 96 hpf using a stereo microscope.

**Table 1:** Selected concentrations of four test substances for metabolome analysis based on lethal concentrations determined through range-finding tests

Treatment substance	Concentration [µM]			Lethal concentration [µM]	
	Low (LTC)	Medium (MTC)	High (HTC)	LC_1_	LC_20_
SU4312	0.5	1.0	1.5	0.58	1.47
Sorafenib	1.5	2.0	2.4	1.95	2.33
Rotenone	0.00625	0.0125	0.025	0.005	0.033
Methotrexate	100	200	250	143	237

The concentration selection was based on the observed LC_1_ and LC_20_ levels in the range-finding tests, such that the highest test concentration (HTC) was close to LC_20_ and the lowest test concentration (LTC) to LC_1_. The medium test concentration (MTC) was selected with appropriate spacing from the other two concentrations. LC_1_ lethal concentration 1%, LC_20_ lethal concentration 20%

A treatment was considered valid if the percentage of non-altered control ZFEs was ≥ 80% and if at least 9/16 ZFEs of each test concentration survived. Only surviving ZFEs were sampled at 96 hpf. 20 solvent control ZFEs, 5 from each treatment plate, and sampled individually, formed the control group. Additionally, 80 solvent control ZFEs were pooled for technical reference during metabolome analyses, hereafter referred to as pool. Samples were frozen in liquid nitrogen and stored at  −80 ℃ until further use.

## Sample preparation and measurement

Samples were freeze-dried applying a temperature (−50 to 30 ℃) and pressure (vacuum, 0.12 mbar, 0.001 mbar) gradient. Metabolites were extracted by adding 500 µL of a mixture of isopropanol and water (80:20 v/v), and by using a ball mill (Bead Ruptor Biolab) for homogenization (3 × 30 s, 4.85 m/s). After centrifugation (15,294×*g*, 10 min, 15 ℃), aliquots of the extract were subjected to LC–MS/MS analysis.

In total, 137 biological samples were measured, along with technical samples to correct for inter- and intra-instrumental variations, and for quality grading. The biological samples comprised 20 control samples, and 8–12 samples from each treatment condition to obtain an adequate number of morphologically unremarkable ZFEs as well as ZFEs with various morphological anomalies. The technical samples comprised ten blanks, ten aliquots of the pool diluted to one ZFE equivalent (100%), and four aliquots each of a dilution series (50, 75, 150, and 200%) of the pool to test the linearity of the feature intensities.

Metabolome analyses were performed in two sequences and on four analytical setups each (lipid and polar phases with positive and negative ionization modes). Biological samples were randomized across the two sequences and the technical samples were repeated.

For lipid reverse-phase high-performance liquid chromatography (RP-HPLC, Ascentis Express C18, 5 cm × 2.1 mm, 2.7 µm, Supelco), 20 µL of extract were injected. RP-HPLC gradient elution was performed with solvent (A) water/methanol/0.1 M ammonium formate (1:1:0.02, w/w), and solvent (B) methyl-tert-butylether/2-propanol/methanol/0.1 M ammonium formate (2:1:0.5:0.035, w/w) with 0.5% (w/w) formic acid (0 min 100% A, 0.5 min 75% A, 5.9 min 10% A; 600 µL/min).

For polar hydrophilic interaction liquid chromatography (ZIC-HILIC, 2.1 × 10 mm, 3.5 µm, Supelco), 4 µL of extract were injected. HILIC gradient elution was performed with solvent (C) acetonitrile/water (99:1, v/v) containing 0.2% (v/v) acetic acid, and solvent (D) 0.007 M ammonium acetate containing 0.2% (v/v) acetic acid (0 min 100% C, 5 min 10% C; 600 µL/min).

Liquid chromatography (Agilent 1290 Infinity) was followed by tandem mass spectrometry on a Q-TOF instrument (AB Sciex TripleTOF 6600) with electrospray ionization (ESI) in positive and negative mode.

## Data processing

### MzMine

Mass spectrometry data for the two measurement sequences were processed together in MzMine (Schmid et al. 2023) for each analytical setup. Signal intensities were exported as maximum peak height and further processed. A feature was defined by its mass and retention time and had different signal intensities for each sample. The following steps were executed: (1) mass detection for MS1/MS2 scans using a noise level of 1000/25 ppm, respectively; (2) feature detection using ADAP chromatogram builder (Myers et al. 2017; Pluskal et al. 2010); (3) deconvolution of chromatograms using ADAP resolver; (4) deisotoping; (5) feature lists alignment; (6) gap filling using the peak finder module; (7) filtering for a minimum of three peaks per feature, gap filling, removal of duplicate features, and filtering for a minimum of five datapoints describing a peak (exact settings in supp.MZsettings.xml).

The Mzmine processing resulted in the following numbers of features per analytical setup: 14,522 for lipid positive, 8366 for polar positive, 5979 for polar negative, and 3610 for lipid negative (32,477 total).

### Filtering and processing

Since untargeted metabolomics aims to detect all metabolites, the sensitivity needs to be sufficiently low, resulting in higher background noise. To eliminate noisy signals and analytical artifacts, and correct for systematic variabilities, meticulous filtering, normalization, and grading steps were applied.

The following data processing steps were executed in R (R Core Team 2022). *Comparing signal to blanks:* (1) Any signal detected in biological or pool aliquots that was lower or equal to the maximum of the corresponding signal for that feature in the blank samples was set to NA. (2) A feature was retained only if the median of the signal intensities for the pool aliquots was at least 2.5 times stronger than the median signal intensity of the blanks (12,088 features remain after this filtering step). *Data normalization:* (3) To correct for inter-instrumental variability, peak intensities were normalized to the respective median intensity of the pool aliquots from the corresponding measurement sequence. As a prerequisite, features had to be present in both sequences and in at least 3/5 pools in each sequence (4604 features remain). *Grading:* (4) The linearity of each feature was determined from the dilution series of 50–200%. Features detected in less than 80% of the linearity samples were excluded (3895 features remain). (5) Based on linearity and relative standard deviation (rsd) of the pools, the quality class was assigned: SQ—when *R*^2^ > 0.64, slope > 0, and rsd < 0.6. Only SQ features were further considered (2123 SQ features remain). (6) A linear model was fitted to the median of pool intensities, comparing the two measurement sequences. Only features that were within the threshold of residual -1 to 1 were retained to remove features with high variability between measurements. (7) Since samples were generated in different weeks, they were batch corrected by normalizing the treatment samples to the median of controls from the same clutch. This required that features were present in at least 8/10 of the controls in the batch (1886 SQ features remain). (8) Features detected in less than 80% of all biological samples were excluded. The final number of features in single ZFE were 1835, including 685 from lipid positive, 440 from polar positive, 409 from lipid negative, and 301 from polar negative setup.

### Data analysis

Initially, a sensitivity test was evaluated comparing samples of ten, two, and one ZFEs in developmental stage 120 hpf (supp. file4 Fig. S1). The number of SQ features was comparable between ten ZFEs (2081), two ZFEs (2195), and one ZFE (2098). Furthermore, the feature count for one ZFE at 120 hpf was comparable to the feature count achieved in the main experiment (2123) measuring one ZFE at developmental stage 96 hpf. Based on these results, the measurement sensitivity was considered sufficient for single-embryo metabolomics.

For the main experiment, the metabolic effects of individual treatments and their shared effects were investigated, using R (R Core Team 2022) and Excel. First, principal component analysis (PCA) was applied to explore the general structure of the entire dataset. Second, for analysis of single feature changes, the ratio between the median intensity of the treated and control samples was calculated (supp.file1). The significance of relative fold changes was assessed using a *t* test; a *p-*value ≤ 0.1 was regarded as significant. Based on the significantly changed features compared to the control, a common effect pattern was identified among the treatment groups SU4312 high, sorafenib medium, rotenone high, and methotrexate medium.

### Feature annotation

Feature annotation was achieved by (a) matching spectral information with GNPS database (Wang et al. 2016), (b) matching of exact mass and retention time with an internal database, and (c) accurate mass matching (details in supp. file4 Table S1) with HMDB (Wishart et al. 2022) and Lipid Maps (Liebisch et al. 2013). Strategy (a) resulted in 36% of annotated features, of which 15% were true matches and 21% analog matches (supp. file2). Strategies (b) and (c) were applied to the common effect pattern of SU4312, sorafenib, and methotrexate and resulted in 83% of annotated features (supp. file3). Annotations were reviewed for biological plausibility.

For the common effect pattern, an annotation score was derived, which indicates how many factors (MS2 spectrum, retention time, mass) were considered. 57% of features achieved an annotation score of 1, 24% a score of 2, and 1% a score of 3. The annotation score and thus the confidence increased, the more information was incorporated.

## Results and discussion

### SU4312, sorafenib, and methotrexate exposed zebrafish embryos show morphological alterations

**Fig. 1:**
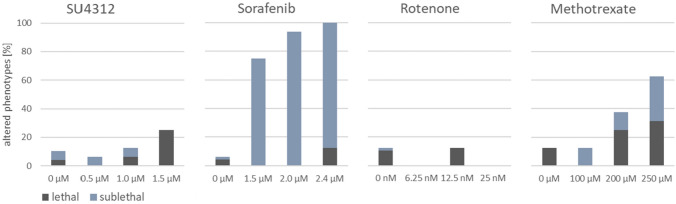
Gross morphological alterations observed for zebrafish embryos (ZFEs) treated with SU4312, sorafenib, methotrexate, or rotenone. Morphological changes were assessed through microscopic inspection of the same embryos that underwent metabolome analysis. SU4312, sorafenib, and methotrexate treatments revealed a concentration-dependent increase in the number of altered phenotypes. For rotenone, no concentration-dependent effects on morphology were observed. Coagulated embryos were classified as lethal, and all other findings as sublethal effects (detailed assessment in supp. file4 Fig. S2)

A concentration-dependent increase in the number of altered phenotypes was observed for SU4312, sorafenib, and methotrexate treatments (Fig. 1) with different manifestations of sublethal effects (supp. file4 Fig. S2). For SU4312, 6% of ZFEs with malformed tail tips were observed each at the lowest test concentration (LTC) and at the medium test concentration (MTC), while no malformations were found at the highest test concentration (HTC). Albeit sorafenib possessed a low lethality of 12.5% at HTC, a high prevalence of severe sublethal effects was observed at all concentrations tested. At HTC, 86% of ZFEs were unhatched. At MTC and LTC, about 80% of the embryos had pericardial edemas. Exposure to methotrexate HTC resulted in 27% of unhatched embryos. Beyond that, malformations and severe retardation were found in about 15% of ZFEs in each test concentration.

The HTCs were selected to correspond to a lethality of 20% in the preliminary range-finding experiment. LC_20_ levels were largely reproduced in the main experiment except for rotenone, which exhibited no lethality at HTC (Fig. 1). The LTCs corresponded to a theoretical lethality of 1%, which should result in no lethality in a group of 16 embryos, as demonstrated in the main experiment. The occurrence of malformations in SU4312, sorafenib, and methotrexate treatments further evidenced that test concentrations were developmentally toxic to ZFE. Since rotenone did not show a continuous increase in phenotypes, the relevant concentration range may not have been met.

HTCs of SU4312, sorafenib, and methotrexate all revealed comparable lethality of approximately 20%, but the number of ZFEs with sublethal effects and their severity differed greatly. The occurrence of edemas and unhatched embryos after exposure to sorafenib, and methotrexate can be considered as severe effects. Test concentrations were selected based on LC values as these are a more robust and reproducible measure between laboratories. Yet, despite the weaker comparability of effective concentrations (ECs), we propose to consider the incidence of severe sublethal effects when selecting test concentrations for metabolomics to avoid measuring highly toxic responses.

### Metabolic and morphological effect levels mirror each other

The evaluation of morphological alterations (Fig. 1) showed that HTCs of sorafenib and methotrexate provoked severe phenotypes, suggesting that HTCs were highly toxic. To identify specific metabolome changes, test concentrations must be high enough to induce sustained biochemical changes, but not too high to avoid induction of overt toxicity. In a study performing metabolomics on HepG2 cells, it was shown that test concentrations in the range of EC_15_ induced specific metabolic responses (Ramirez-Hincapie et al. 2023). Concentrations equivalent to EC_85_ induced nonspecific responses representative of general cytotoxicity. Since ECs are not readily transferrable from cell culture to ZFEs, we analyzed feature changes at each test concentration to find evidence of specific and nonspecific metabolome responses in ZFE.

**Fig. 2:**
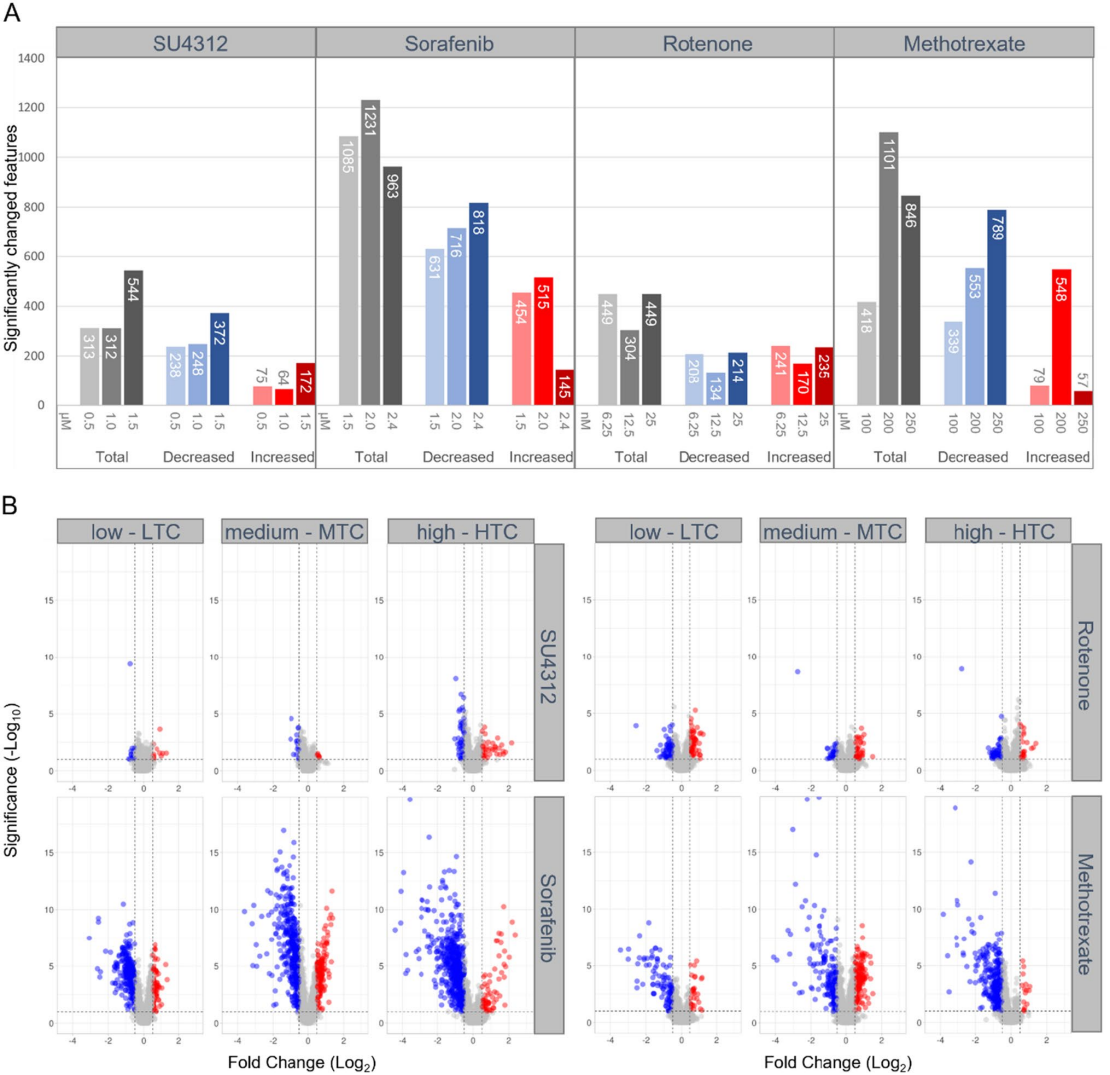
Quantity and intensity of significantly changed LC–MS features measured in zebrafish embryos exposed to SU4312, sorafenib, rotenone, or methotrexate in three (low, medium, high) concentrations each. The total number of features included in the analysis was 1835. **A** Number of significantly (*p* ≤ 0.1) changed features per experimental condition. Increasing color intensity indicates the increasing applied concentration. Gray bars depict the total number of significant changes, while blue and red bars depict significant decreases and increases, respectively. **B** Volcano plots illustrate relative fold changes and their significance. Red dots indicate significantly increased features, while blue dots indicate significantly decreased features. *LTC* lowest test concentration, *MTC* medium test concentration, *HTC* highest test concentration

Throughout the paper, we only consider the metabolome changes that were statistically significant at a *p*-value ≤ 0.1; for brevity, we omit the word significant. Sorafenib and methotrexate treatments revealed a rise in the total number of changed features from LTC to MTC, followed by a drop from MTC to HTC (Fig. 2A). This was also observed for the number of increased features, while the number of decreased features revealed a continuous concentration-dependent rise. The overlap of changed features was greatest between LTC and MTC of sorafenib (supp. file4 Fig. S3A, B). For methotrexate, the overlap of increased features was greatest between LTC and MTC, while for decreases, the greatest overlap was found between MTC and HTC. SU4312 revealed the most changes with 544 at HTC (Fig. 2A). The overlap of changed features was greatest between MTC and LTC (supp. file4 Fig. S3A, B). For rotenone, an equal number of features were changed in LTC and HTC, while fewer changes were measured at MTC (Fig. 2A). The overlap of changed features was greatest between LTC and HTC (supp. file4 Fig. S3A, B).

The volcano plots illustrate relative fold changes of individual features per experimental condition (Fig. 2B). The metabolic effect levels of sorafenib and methotrexate exposures were higher in terms of significance and magnitude than those of SU4312 and rotenone. Additionally, at HTCs of sorafenib and methotrexate, an imbalance between decreased and increased features was noted in favor of the decreased.

The observations made in the univariate analysis reflected the morphological effect levels in terms of magnitudes and trends. For SU4312, the strongest effects were noted with HTC for both morphology and individual feature changes. Between LTC and HTC of rotenone, the number of feature changes, as well as the morphological effect levels were on par. Lethality only occurred in rotenone MTC and, simultaneously, a lower number of changed features, which might be explained by a higher within-group variability. In sorafenib and methotrexate treatments, the incidence of altered phenotypes was markedly higher compared to rotenone and SU4312, and likewise the number of changed features. The drop in the number of increases in HTCs may be explained by the incidence of unhatched and severely retarded embryos. Hatching usually occurs between 48 and 72 hpf (Kimmel et al. 1995). Hence, unhatched zebrafish at 96 hpf were delayed in development. The drop of increased features and the rise of decreased features suggested that the mass balance was affected, which might impede the detection of specific metabolome alterations. Additionally, the overlap of changed features was greatest for MTCs, supporting that metabolome alterations were more consistent in MTCs than in HTCs. Thus, considering the quantity of feature changes, the overlap of changes between concentrations, and the occurrence of severe morphological alterations, MTCs of sorafenib and methotrexate, and HTCs of SU4312 and rotenone were assessed to reveal a specific MoA.

### Zebrafish embryos with abnormal morphology show a divergent metabolic response

**Fig. 3:**
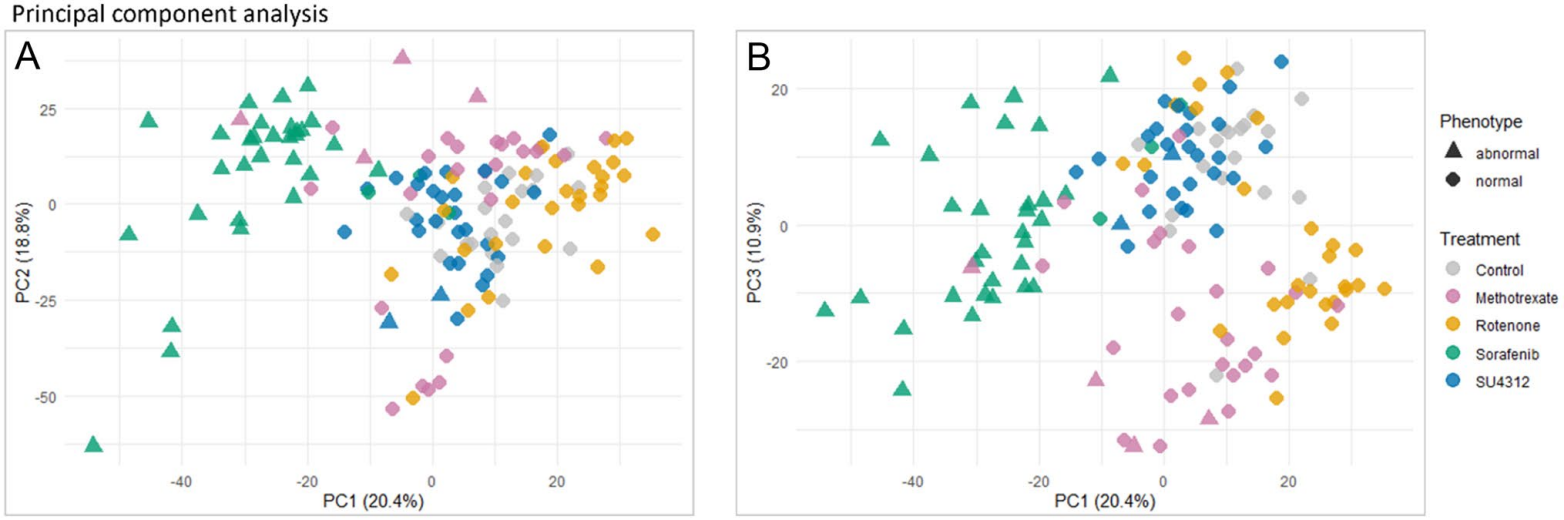
Principal component analysis (PCA) representing metabolic profiles of individual zebrafish embryos treated with SU4312, sorafenib, rotenone, or methotrexate in three concentrations each. Phenotype assessment and untargeted metabolomics were performed on the same individual. A cumulative metabolic variance of 50% is described by the first three principal components (PCs), with 20.4% by PC1, 18.8% by PC2 (**A**), and 10.9% by PC3 (**B**). (PCA with labeled test concentrations is shown in  supp. file4 Fig. S4)

PCA is an unsupervised multivariate statistical approach to explore the general structure of a high-dimensional dataset by reducing its dimensionality while preserving the most relevant component of the information. A cumulative variance of 50% was captured by the first three principal components (Fig. 3). The main separation of samples in PC1/PC2 (Fig. 3A) was driven by the occurrence of abnormal phenotypes. Among the sorafenib-treated ZFEs, three embryos without visible morphological alterations tended to cluster with the controls. Of the methotrexate exposed ZFEs with abnormal phenotype, two embryos clearly clustered with the sorafenib phenotypes, while another two showed a slight separation from this group. The former two were severely retarded and overall malformed. The latter two were malformed, though no retardation was observed. From SU4312 treatment, two ZFEs with malformed tail tip did not cluster with the abnormal phenotypes.

To check for systematic effects in higher PCs, PC1/PC3 were evaluated (Fig. 3B). Two main trends were observed. First, a separation of samples in the diagonal of PC1/PC3 was noted. This was mainly seen for sorafenib and SU4312; however, methotrexate samples also separated in parallel along this axis. Second, a separation in the orthogonal direction of the diagonal was perceived. Mainly rotenone samples owned this trend, yet, a separation in the orthogonal direction was also inherited by methotrexate samples. Thus, the angiogenesis inhibitors, SU4312 and sorafenib, seemed to share metabolic effects with methotrexate but not with rotenone (see also supp. file4 Fig. S5). Rotenone seemed to share some metabolic effects with methotrexate.

PC1/PC2 suggested that ZFEs possessing signs of severe toxicity like edema or retardation were metabolically distinct from those with no or mild morphological alterations. It is unclear whether the metabolic changes caused the altered morphology, or if alterations in morphology affected the composition of embryos and thus changed the metabolite composition, or if both factors contributed.

PC1/PC3 revealed two trends in orthogonal directions, suggesting two different underlying MoAs. (1) The separation of sorafenib, SU4312, and methotrexate samples on the diagonal indicated shared metabolic effects potentially related to antiangiogenesis. SU4312 and sorafenib both are specific inhibitors of angiogenesis and share VEGFR2 as a primary target (Sun et al. 1998; Wilhelm et al. 2006). Yet, apart from VEGFR2 signaling, many other molecular modulators, such as HIF-1 (hypoxia-inducible factor 1), FGF (fibroblast growth factor) or Notch signaling, steer angiogenesis (Andersson et al. 2011; Presta et al. 2005; Zimna and Kurpisz 2015). Methotrexate has been suspected to impair vascular development (Joussen et al. 1999; Schoors et al. 2015; Sun et al. 2009), but the underlying mechanism remains obscure. (2) The separation of rotenone and methotrexate samples in orthogonal directions indicated that they share common metabolic effects. These common effects may be traced back to disturbances in the mitochondrial respiratory chain leading to decreased energy availability (Kolli et al. 2014; Palmer et al. 1968). In summary, PC1/PC3 illustrated that methotrexate shared metabolic effects with SU4312 and sorafenib, which might be related to antiangiogenesis, and with rotenone, which might be related to energy metabolism.

### Methotrexate shares significant metabolic changes with specific inhibitors of angiogenesis

**Fig. 4:**
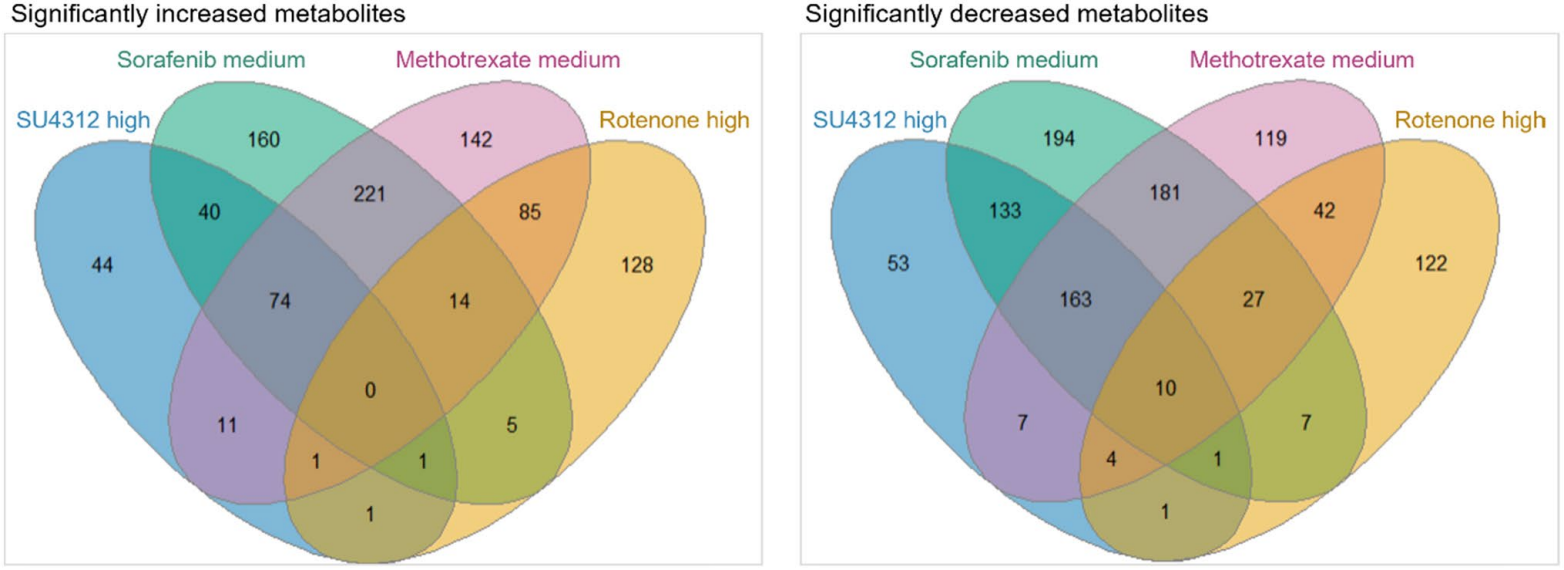
Overlap of increased/decreased LC–MS features among SU4312, sorafenib, methotrexate, and rotenone treated zebrafish embryos. The high concentrations of SU4312 and rotenone, and the medium concentrations of sorafenib and methotrexate were analyzed with respect to common metabolome changes. Test groups were selected based on morphological findings and metabolic effect levels (Figs. 1, 2). No common features were found to be significantly (*p* ≤ 0.1) increased among all test groups. 74 features were commonly increased among SU4312, sorafenib, and methotrexate treatments. 10 features were significantly decreased across all test groups, and 173 when excluding rotenone (for statistical analysis of intersections, see supp. file4 Fig. S3C).

For the test concentrations of SU4312, sorafenib, and methotrexate treatments selected based on evidence of a specific response, an overlap of 74 commonly increased and 173 commonly decreased features was observed (Fig. 4). Including rotenone, ten shared decreased features were found across all four treatments, though this intersection was not statistically significant (supp. file4 Fig. S3C).

Methotrexate has been linked to several MoAs, which could explain the variety of congenital malformations found in humans (Verberne et al. 2019). The complete mechanism of toxicity remains elusive, partly due to conflicting findings, including its role in angiogenesis. In this study, a total of 247 jointly changed features were found among the specific angiogenesis inhibitors, SU4312 and sorafenib, and methotrexate. This consensus strongly suggested that methotrexate acts through an antiangiogenic MoA. Similar to methotrexate, rotenone was connected to a multitude of MoAs. Different in vitro tests have classified rotenone as a putative vascular disruptor (McCollum et al. 2017). However, no evidence of a shared MoA with SU4312 and sorafenib was detected in the metabolic signature of rotenone, which would corroborate an antiangiogenic action of rotenone. As the aspired LC_20_ level was not reached for rotenone HTC, further analysis of higher concentrations is warranted to investigate potential antiangiogenic properties.

**Fig. 5:**
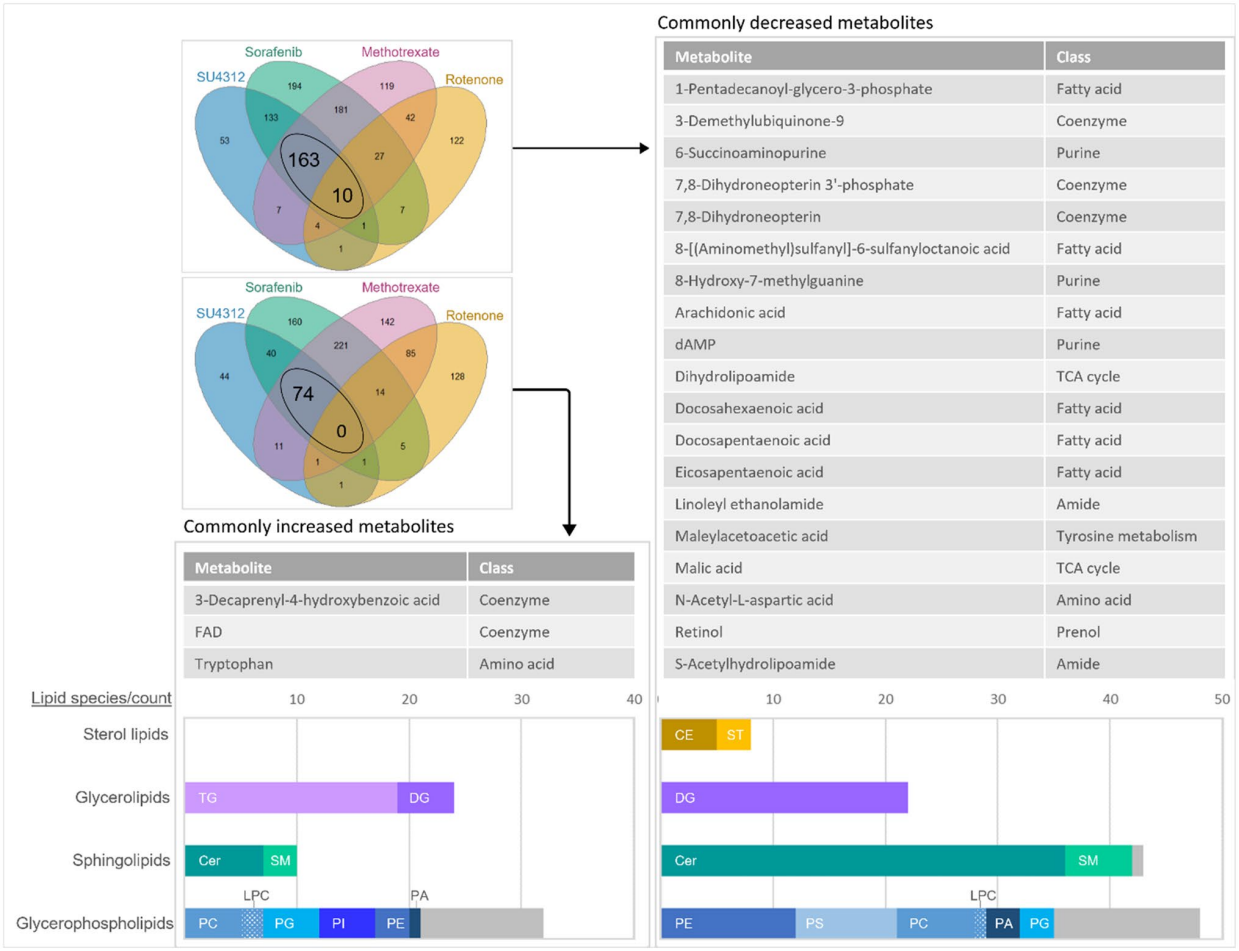
Identity of commonly changed LC–MS features of SU4312, sorafenib and methotrexate treatments. Spectral information, exact mass, and retention time were used to annotate 247 commonly changed features (detailed list including annotation score in supp. file3). 83% of features were annotated, these are shown. Lipids were summarized and grouped based on their (sub-)class. Apart from lipids, several fatty acids, coenzymes, and other essential metabolites were identified. *CE* cholesteryl ester, *Cer* ceramide, *DG* diglyceride, *LPC* lysophosphatidylcholine, *PA* phosphatidic acid, *PC* phosphatidylcholine, *PE* phosphatidylethanolamine, *PG* phosphatidylglycerol, *PI* phosphatidylinositol, *PS* phosphatidylserine, *SM* sphingomyelin, *ST* sterol, *TG* triglyceride

The pattern of 247 commonly changed features among SU4312, sorafenib, and methotrexate consisted of 75% lipids from various classes and subclasses. Since the representatives of a subclass can be readily converted into each other, they were grouped accordingly. Among the class of glycerophospholipids, some subclasses were represented in both the increased and decreased set, such as PC, PE, PG, and PA (Fig. 5). Other subclasses were exclusively found in the increased (PI, TG) or decreased (PS, CE, ST) set. Most decreased fatty acids belonged to the class of very long chain n-3 and n-6 polyunsaturated fatty acids, such as arachidonic acid, docosahexaenoic acid, docosapentaenoic acid, and eicosapentaenoic acid. Furthermore, several coenzymes and representatives of key metabolic pathways such as TCA cycle, amino acid, and purine metabolism were found in the common pattern of SU4312, sorafenib, and methotrexate.

Phosphatidylinositols (PI) are gradually phosphorylated to different phosphoinositides, such as phosphatidylinositol (4,5)-bisphosphate (PIP2) and phosphatidylinositol (3,4,5)-trisphosphate (PIP3), which act as important signaling molecules (Czech 2000). The initial phosphorylation of PI is mediated by the phosphatidylinositol 4-kinases (Pi4k). Downregulation of Pi4k in ZFE caused severe developmental defects with abnormal pectoral fins being the most prominent (Ma et al. 2009). This may be explained by disturbances of the Pi3k (PIP2 3-kinase)–Akt signaling pathway. Pi3k phosphorylates PIP2 to generate PIP3, which is a key regulator of fundamental cellular processes including cell proliferation and angiogenesis. Thus, impairments of PI downstream signaling could cause developmental defects via disruption of angiogenesis. The accumulation of PI may be considered as an indicator for impairments of PI downstream signaling.

Additionally, diglycerides (DG) should be considered in the context of PI downstream signaling. While PI were increased, the vast majority of DG were decreased. DG are employed in the regeneration of phosphoinositides (Antonsson 1997). A reduction in DG may reflect impairments of PI downstream signaling. However, DG can also be synthesized directly from PI through phospholipase C, which could raise DG levels in the presence of elevated PI levels. Though most DG were decreased, we also measured increased DG species, suggesting the involvement of diverse DG species in various pathways. Since there is currently limited knowledge about the contribution of certain DG species to distinct pathways, their potential as indicators of antiangiogenesis and developmental toxicity remains questionable.

Representatives of triglycerides (TG) were found exclusively in the set of increased metabolites. Fraher et al. (2016) have shown, that while DG increased in body tissue during embryonic development, TG levels remained at a constantly low level. In contrast, in the yolk, DG levels remained stable while TG levels decreased over time. This emphasized the distinct roles of TG and DG. While DG are involved in signaling cascades, TG mainly serve as an energy reservoir. Elevated TG levels could therefore indicate that less yolk was consumed potentially as a consequence of impaired or delayed development.

Phosphatidylserines (PS) are known to play a vital role in brain development and angiogenesis. PS are crucial apoptotic markers recognized by BAI (brain-specific angiogenesis inhibitor) transmembrane receptors, which are predominantly expressed in brain tissues (Park and Ravichandran 2010). The extracellular domain of BAI, accountable for recognition of PS and conveying phagocytosis, has a second function in inhibiting angiogenesis. Little is known about antecedents of BAI-mediated antiangiogenesis. Presumably, lower PS levels could enable the BAI extracellular domain to exert its second function, which is inhibition of angiogenesis. Moreover, in zebrafish embryonic development, PS seem to own a special role. Among the phospholipids, the prevalent subclasses PC, PE, and PI are present in equal amounts in the body as in yolk of 6 hpf ZFE, however, the relative abundance of PS is significantly higher in the body (Castanon et al. 2020). The function of PS in vertebrates has not been fully elucidated. Yet, there is evidence that PS can regulate angiogenesis via BAI, which might be of special importance for brain development.

Cholesteryl esters (CE), which were decreased, are the dominant transport and storage form of cholesterols that are essential components of cell membranes. Lyu et al. (2019) established a link between depletion of membrane cholesterol and suppression of angiogenesis via inactivation of mTor signaling in human endothelial cells, and ZFE. Another mechanistic link between cholesterol depletion and VEGFR2-dependent inhibition of angiogenesis was provided by Fang et al. (2013) via the regulation of cholesterol efflux. These findings from in vitro and ex vivo systems necessarily suggest a correlation of diminished cholesterol and disturbed angiogenesis. We advocate that decreased CE levels are concomitant with lower cholesterol levels, which may be a key indicator of mTor/VEGFR2-mediated antiangiogenesis.

Furthermore, there is compelling evidence for fatty acids to be highly involved in angiogenesis. Inhibition of the fatty acid β-oxidation rate-limiting enzyme decreased vascular sprouting in vivo and in vitro via reduced endothelial cell proliferation (Schoors et al. 2015). Despite the high energy demand, reduced proliferation does not appear to result from a diminished supply of energy equivalents by fatty acids. Instead, it has been suggested that endothelial cells (ECs) possess an exceptional characteristic, wherein they rely on fatty acids as carbon source for the synthesis of deoxyribonucleotides. Apart from decreased fatty acids we also found decreased purines. Our results agree with Schoors et al. (2015) and promote a causative link between decreased fatty acids and reduced angiogenesis via decreased purine deoxyribonucleotides. Furthermore, the reduced n-3 fatty acids might also relate to reduced PS. Hamilton et al. (2000) compiled this connection by showing that reduced intake of n-3 fatty acids during pre- and postnatal development leads to a decrease of n-3 fatty acids and PS in rat brain. While some changes in fatty acids might be specific indicators of antiangiogenesis, changes in overall fatty acid composition might also represent an adaptive response to interference with membrane fluidity (Hachicho et al. 2015).

Intriguingly, the pattern of commonly changed metabolites includes increased 3-decaprenyl-4-hydroxybenzoic acid (DHB) and decreased 2-demethylubiquinone-9 (DeMQ). Both metabolites are intermediates of the ubiquinone biosynthesis. While DHB is located at the beginning of the pathway, DeMQ is the direct precursor of the end product coenzyme Q (CoQ). The substance treatments could interfere with downstream enzymes of DHB resulting in lower levels of DeMQ, accumulation of DHB, and potential deficiency of CoQ. CoQ deficiency has been described to interfere with the development of various organs including the brain and cardiovascular system in mammals (Alcázar-Fabra et al. 2021; Lu et al. 2012). Cardiovascular defects were also found in zebrafish mutants with depleted CoQ levels accompanied by increased reactive oxygen species and lipid peroxidation in vascular cells (Mugoni et al. 2013). Another aspect to consider is that DHB contains 10 isoprene residues, while DeMQ contains 9, making it a precursor of CoQ9, whereas DHB is a precursor of CoQ10. Most species possess a dominant CoQ, which in zebrafish and humans is CoQ10, although non-dominant CoQs have also been detected (Kawamukai 2009). Still, further research is needed to clarify the role of non-dominant CoQ species in ZFEs. DeMQ could possibly serve as an indicator of CoQ deficiency, which is linked to developmental disorders.

Pterine and purine metabolic pathways are tightly connected to the folate biosynthesis. The purine metabolism provides GTP and the measured pterines are biochemical precursors of folate. There is ample evidence that folate deficiency hinders neural tube closure, emphasizing the need for preventive supplementation of folate during pregnancy (Ross 2010). In an effort to disentangle the mechanism of folate-dependent neural tube defects, it was observed that decidual angiogenesis was decreased in pregnant folate-deficient mice as well as proangiogenic factors like VEGFR2 (Li et al. 2015). All measured folate biosynthesis-related metabolites were decreased, supporting a lack of folate was lacking, which might be causative or concomitant in antiangiogenesis.

Apart from folate, retinoids have long been recognized as important regulators of embryogenesis, including development of the limbs, brain, and heart (Hill et al. 1995; Osmond et al. 1991; Tickle et al. 1982). It was observed that retinoids can have an inhibitory effect on angio- and vasculogenesis (Hoffmann et al. 2007; Oikawa et al. 1989; Pawlikowski et al. 2019). In zebrafish, retinol levels start to rise around 48 hpf under physiological conditions (Costaridis et al. 1996). The exposure to SU4312, sorafenib, and methotrexate all independently caused a 50% reduction of retinol levels in comparison to controls (supp. file3). This profound decrease in retinol levels might be an important indicator of developmental impairments.

## Conclusion

Using single-embryo analysis, we identified a common metabolic effect pattern of SU4312, sorafenib, and methotrexate treatments in zebrafish. The concordance of 247 metabolic changes between two specific inhibitors of angiogenesis, SU4312 and sorafenib, and the teratogen methotrexate suggests a common MoA associated with developmental toxicity and antiangiogenesis. The specificity of the metabolome response for antiangiogenesis and developmental toxicity was supported by decreased levels of retinol, CoQ precursors, phosphatidylserines, cholesteryl esters, fatty acids, purines, and pterines, as well as increased levels of phosphatidylinositols. The metabolome response of rotenone, a known inhibitor the mitochondrial respiratory chain, differed from SU4312 and sorafenib. However, the higher similarity to methotrexate indicates that both substances may disturb energy metabolism.

Single-embryo untargeted metabolomics allowed for a direct correlation between morphological and metabolic phenotypes, which mutually influence each other as demonstrated in the PCA. The number of features was augmented by a factor of five compared to our targeted approach (Wilhelmi et al. 2023). Yet, a major challenge in untargeted metabolomics resides in the annotation process. For the pattern of 247 metabolites, we achieved an annotation rate of 83% by utilizing different feature characteristics. The bottleneck in developing the approach into a screening system is currently the annotation via accurate mass matching, which could not be fully automated.

Moreover, the selection of test concentrations plays a pivotal role in elucidating a specific metabolic effect pattern. We previously applied a concentration–response modelling and point-of-departure approach to determine the test concentration depicting a specific response (Wilhelmi et al. 2023). For a robust modeling, however, at least five test concentrations are required, which ideally include nonspecific responses to fit the lower and upper asymptote of the response curve. Measuring nonspecific metabolome responses is not economical, and reducing the number of test concentrations is a crucial step in developing a high-throughput screening assay. Here, we have shown that the relevant test concentration can be identified by considering morphological alterations and individual feature changes. The relevance of metabolic changes was demonstrated by the overlap between different substances. We recommend dedicating further efforts to concentration range-finding experiments and considering the EC in addition to the LC, especially when encountering severe phenotypes. The HTC should not exceed 20% of lethality.

It was demonstrated that single-embryo untargeted metabolomics is able to identify substance- and MoA-specific metabolome responses in zebrafish. The detected metabolic effect pattern can help identifying biomarkers to advance the prediction of developmental toxicity based on an assay free from animal tests. Some metabolite changes were associated with antiangiogenesis, these could contribute to enhance the comprehension of antiangiogenesis-related developmental toxicity. Still, the specificity of metabolite changes for antiangiogenesis and developmental toxicity should be reinforced by comparison with another MoA of developmental toxicity, such as the induction of a cleft palate or by functional genetic studies.

### Supplementary Information

Below is the link to the electronic supplementary material.

Supplementary file1 (XLSX 646 kb)

Supplementary file2 (XLSX 272 kb)

Supplementary file3 (XLSX 108 kb)

Supplementary file4 (DOCX 917 kb)

## Data Availability

Data will be made available upon reasonable request.
