# Supplementary material for: Molecular signatures of angiogenesis inhibitors: a single-embryo untargeted metabolomics approach in zebrafish
Source: Arch Toxicol. 2024 Jan 29;98(3):943–56. doi: 10.1007/s00204-023-03655-5 (PMC10861732; doi:10.1007/s00204-023-03655-5)
Supplement: Supplementary file 4 — Supplementary file4 (DOCX 917 kb) [file 204_2023_3655_MOESM4_ESM.docx]

# SUPPLEMENTARY

**Table S1** Settings used for accurate mass matching with HMDB and LipidMaps databases.

| **Setup** | **Adduct Type** | **Molecular Weight Tolerance** |
| --- | --- | --- |
| Lipid positive | M+H  M+H-H2O  M+NH4  M+Na | 0.005 Da |
| Lipid negative | M-H  M+FA-H | 0.005 Da |
| Polar positive | M+H  M+H-H2O  M+NH4  M+Na | 0.005 Da |
| Polar negative | M-H  M+Hac-H | 0.005 Da |

**Table S2** Morphological endpoints assessed by visual inspection.

| **Morphological endpoints assessed** | |
| --- | --- |
| c | coagulated |
| unh | unhatched |
| noeye | no formation of eyes |
| n/l pig | now/low pigmentation |
| edY/edP | edema yolk/pericard |
| ret | retardation |
| mhe | malformation head |
| meye | malformation eye |
| mtail | malformation tail |
| mtip | malformation tip of tail |
| sco | scoliosis |
| omal | overall malformed |

**
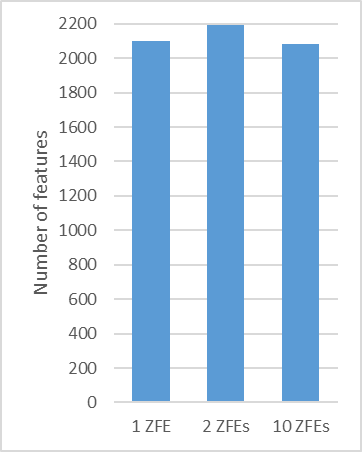
**

**Fig. S1** Sensitivity test to assess the potential loss of features when reducing the number of zebrafish embryos (ZFEs) per sample. The number of SQ features measured in an untargeted metabolomics approach for 1, 2, or 10 zebrafish embryos (ZFEs) at developmental stage 120 hours post fertilization (hpf). Five replicates each were measured and only features present in all replicates were considered.


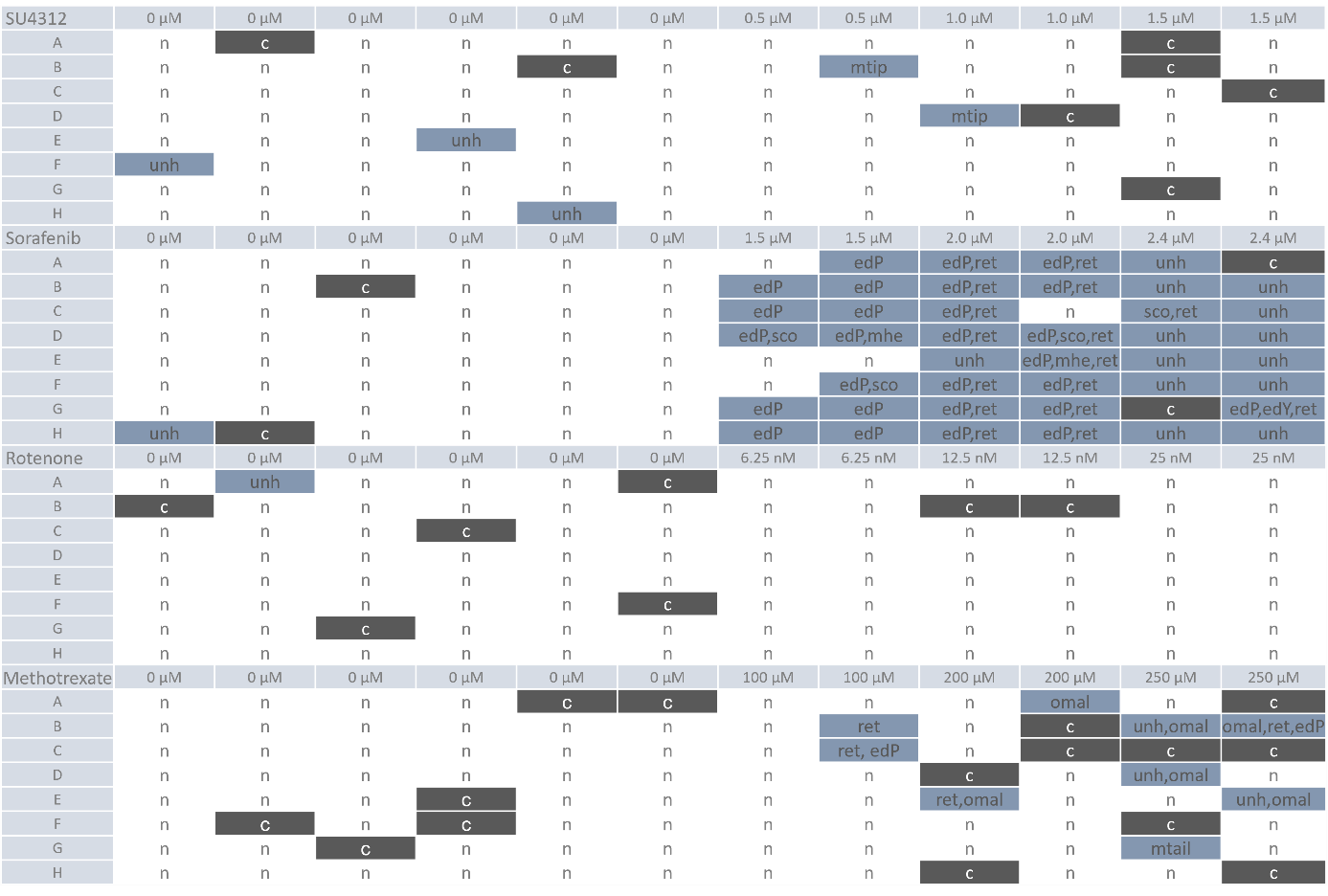


**Fig. S2** Plate layout and morphological findings of zebrafish embryos treated SU312, sorafenib, rotenone, or methotrexate in three concentrations each. Solvent controls, exposed to 0.001% DMSO, are indicated as 0 µM.

c - coagulated; edP - edema pericard; edY - edema yolk; mhe - malformation head; mtail - malformation tail; mtip - malformation tip of tail; n - normal; ret – retarded; omal - overall malformed; sco - scoliosis; unh - unhatched

**
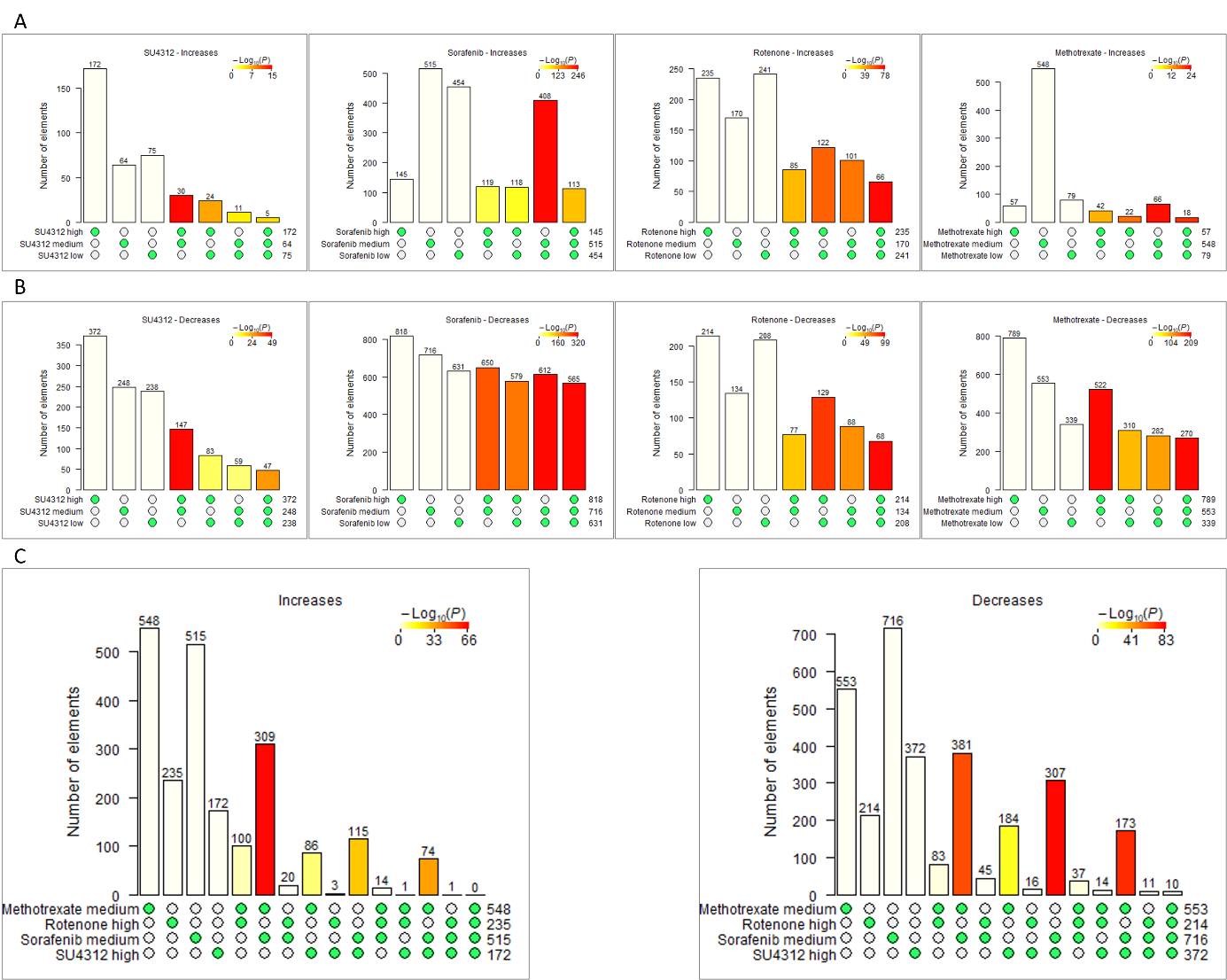
**

**Fig. S3** Significance of overlap of changed features between test groups. The number of elements refers to the number of features jointly changed among test groups. For multi-set intersection analysis, the R package ‘SuperExact Test’ was used (Wang et al. 2015). **A**) Overlap of significant (p ≤ 0.1) increases among three test concentrations of a test substance. **B**) Overlap of significant decreases among three test concentrations of a test substance. **C**) Overlap of significant increases/decreases among all test substances for selected concentrations.


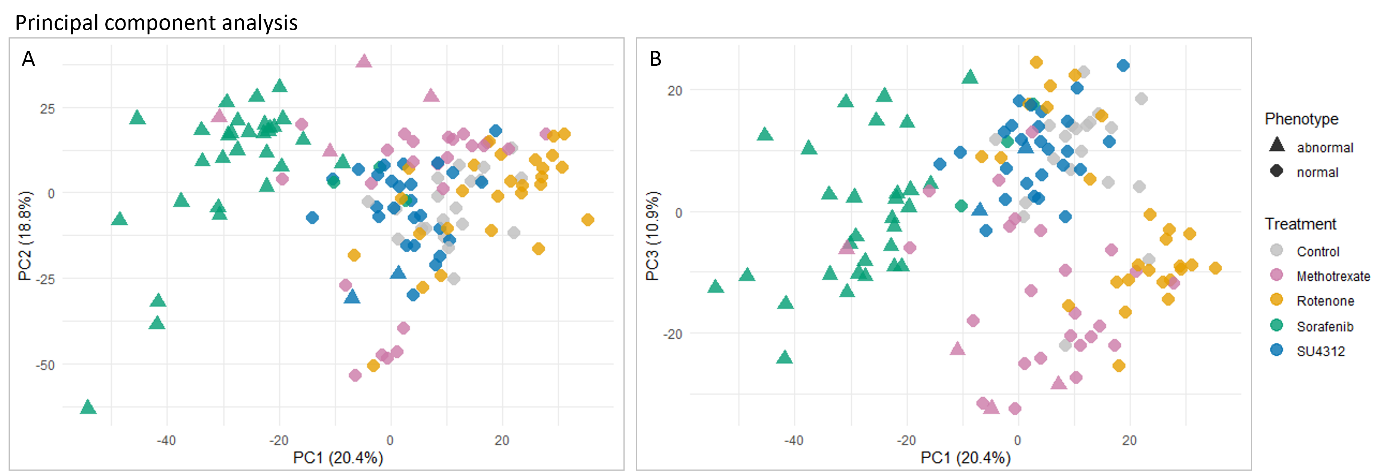


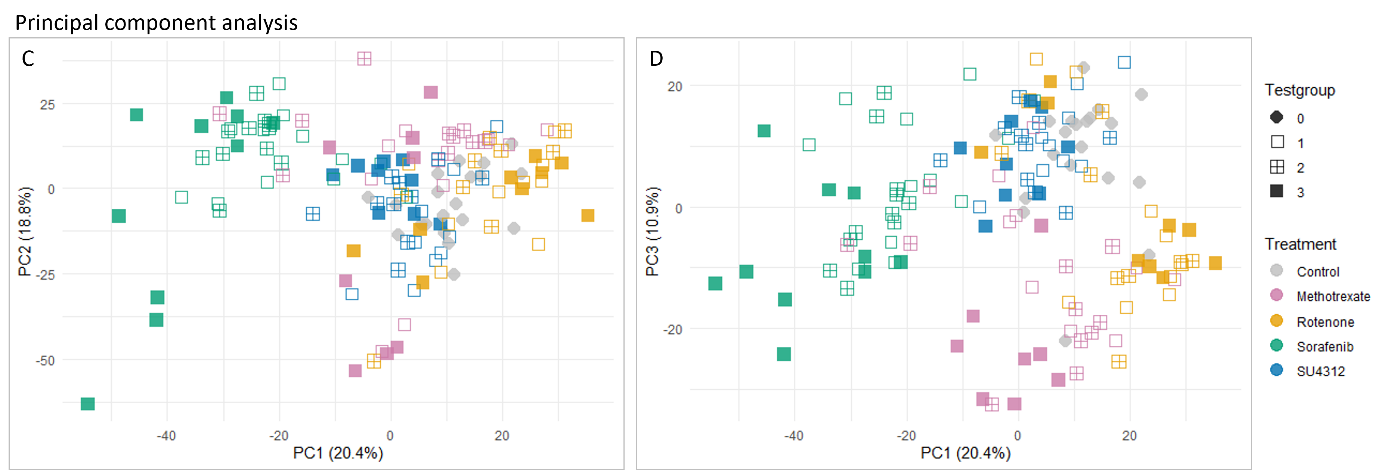


**Fig. S4** Principal component analysis representing metabolic profiles of individual zebrafish embryos (ZFE) treated with either methotrexate, rotenone, sorafenib, or SU4312 in three concentrations each. **A**) PC1 vs. PC2 and **B**) PC1 vs. PC3 illustrating abnormal phenotypes; **C**) PC1 vs. PC2 and **D**) PC1 vs. PC3 illustrating different concentrations with 1 - lowest test concentration, 2 - medium test concentration, and 3 - highest test concentration


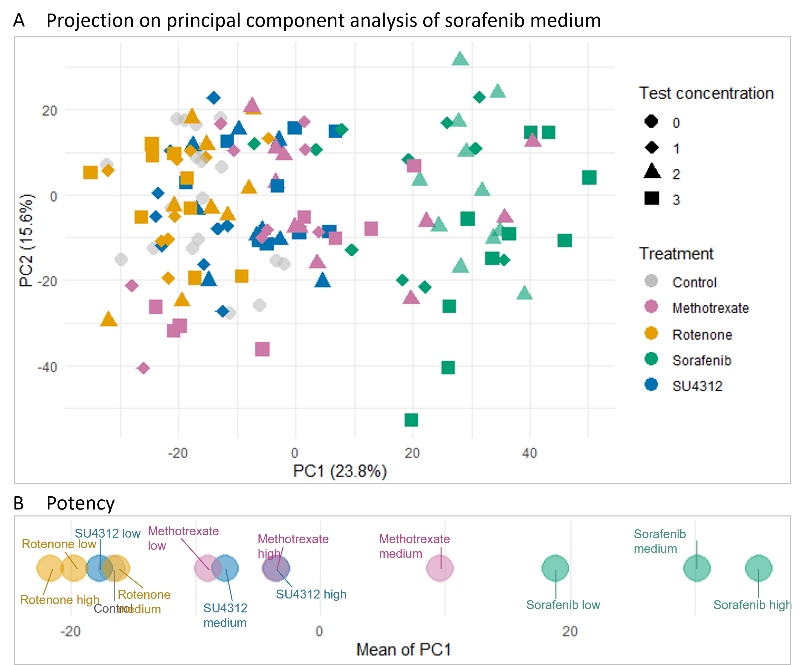


**Fig. S5** Inheritance and potency of metabolic response of sorafenib medium by remaining samples. **A**) Sorafenib medium was used as a training data set to compute a principal component analysis (PCA). The remaining samples were projected onto the PCA to visualize how the metabolic response of sorafenib is adopted by them. **B**) The potency of the inherited sorafenib response was estimated by comparing the mean of PC1 values per test concentration. 0 - control, 1 - lowest test concentration, 2 - medium test concentration, and 3 - highest test concentration
